# Lipidomic and transcriptomic profiles of glycerophospholipid metabolism during *Hemerocallis citrina* Baroni flowering

**DOI:** 10.1186/s12870-022-04020-x

**Published:** 2023-01-23

**Authors:** Aihua Guo, Yang Yang, Jiang Wu, Nannan Qin, Feifan Hou, Yang Gao, Ke Li, Guoming Xing, Sen Li

**Affiliations:** 1 Department of life science, Lyuliang University, Lvliang, 033000 China; 2 College of Horticulture, Shanxi Agricultural University, Taigu, 030801 China; 3 Datong Daylily Industrial Development Research Institute, Datong, 037000 China

**Keywords:** *Hemerocallis citrina* Baroni, Lipidomics, Transcriptomics, Glycerophospholipids

## Abstract

**Background:**

*Hemerocallis citrina* Baroni (daylily) is a horticultural ornamental plant and vegetable with various applications as a raw material in traditional Chinese medicine and as a flavouring agent. Daylily contains many functional substances and is rich in lecithin, which is mostly composed of glycerophospholipids. To study the comprehensive dynamic changes in glycerophospholipid during daylily flowering and the underlying signalling mechanisms, we performed comprehensive, time-resolved lipidomic and transcriptomic analyses of ‘Datong Huanghua 6’ daylily.

**Results:**

Labelling with PKH67 fluorescent antibodies clearly and effectively helped visualise lipid changes in daylily, while relative conductivity and malonaldehyde content detection revealed that the early stages of flowering were controllable processes; however, differences became non-significant after 18 h, indicating cellular damage. In addition, phospholipase D (PLD) and lipoxygenase (LOX) activities increased throughout the flowering process, suggesting that lipid hydrolysis and oxidation had intensified. Lipidomics identified 558 lipids that changed during flowering, with the most different lipids found 12 h before and 12 h after flowering. Transcriptome analysis identified 13 key functional genes and enzymes in the glycerophospholipid metabolic pathway. The two-way orthogonal partial least squares analysis showed that diacylglycerol diphosphate phosphatase correlated strongly and positively with phosphatidic acid (PA)(22:0/18:2), PA(34:2), PA(34:4), and diacylglycerol(18:2/21:0) but negatively with phospholipase C. In addition, ethanolamine phosphotransferase gene and phospholipid-N-methyltransferase gene correlated positively with phosphatidylethanolamine (PE)(16:0/18:2), PE(16:0/18:3), PE(33:2), and lysophosphatidylcholine (16:0) but negatively with PE(34:1).

**Conclusions:**

Overall, this study elucidated changes in the glycerophospholipid metabolism pathway during the daylily flowering process, as well as characteristic genes, thus providing a basis for future studies of glycerophospholipids and signal transduction in daylilies.

**Supplementary Information:**

The online version contains supplementary material available at 10.1186/s12870-022-04020-x.

## Background

*Hemerocallis citrina* Baroni, also known as daylily, is a perennial monocotyledon that is used as an ornamental flower in China [1], edible vegetable, ingredient in traditional Chinese medicine, and a flavouring agent [2]. Furthermore, daylilies have considerable economic value and potential applications [3] due to the presence of various functional components, including amides, polyphenols, anthraquinone, flavonols, naphthol [4], alkaloids [5], rutin, and zeaxanthin [3]. Daylily is also rich in lecithin, which is mostly composed of glycerophospholipids [6]. Pharmacological studies have revealed that daylilies can exert antidepressant, anti-inflammatory, and antitumour effects and have explored the main functional factors and signalling pathways underlying these effects [7–9]. For instance, phenolic extracts of daylily have been shown to increase the concentration of neurotransmitters (5-hydroxy tryptamine, dopamine, and norepinephrine) in the hippocampus and frontal cortex via the nuclear factor kappa B signalling pathway, which is inhibited by antidepressants [7, 8]. As daylilies bloom for just 24 h from bud opening to flower tissue wilting, they are often used as a model to study the circadian rhythm of flowers [10, 11], programmed cell death, and flower senescence [12, 13]. Yang et al. [14] studied the pectin features and cell wall changes in daylily tepals during flower opening and senescence and revealed that remodelling of the cell wall pectin is necessary for the occurrence of senescence. Qing et al. [1] provided the first chromosome-level genome of *Hemerocallis citrina* Borani that provides new insights into rutin biosynthesis and lack of colchicine. Twenty-three key circadian clock genes were identified, which related to sensitivity to light signal input and gating, and these genes might closely relate to Flower opening time (FOT) in Hemerocallis [10].

During plant senescence, membrane deterioration is commonly associated with the progressive decrease in the membrane phospholipid content. Changes in phospholipase D (PLD) activity have been observed in many physiological processes, including senescence [15]. For instance, the loss of membrane integrity during petal senescence in *Tradescantia* sp. has been associated with a significant decrease in phospholipid content [16]. In addition, plant senescence is characterised by the degradation of cell membranes, and one of the most characteristic features of membrane degradation is the massive decline in phospholipid content [17]. Biochemical changes in cell membranes during senescence include a simultaneous decrease in the contents of all classes of phospholipids and an increase in that of neutral lipids, and the senescence of carnation flowers is accompanied by increased lipid peroxidation and fatty acid de-esterification [18]. Studies on the senescence of roses revealed that the decline in phosphatidylcholine (PC) content with age was due to its lower biosynthetic capacity in older petals and not due to its increased degradation. The decrease in PC content with age paralleled the overall decline in the content of phospholipids, and cytidine-diphosphocholine (CDP-choline) diacylglycerol phosphorylcholine phosphotransferase (CDP-choline phosphotransferase), which is involved in PC synthesis, might be related to such changes [19]. Triacylglycerol content, along with the content of 60 other lipases, has been reported to increase during natural senescence of Arabidopsis leaves [20]. Bax inhibitor-1 is regarded as a cell death suppressor that can interact with other molecules to alter lipid dynamics [21]. In Arabidopsis, leaf senescence-related 1 may be involved in the transport of specific lipid signalling molecules to regulate senescence in leaves [22].

Omics-based technologies, such as transcriptomics, proteomics, and metabolomics, have recently been used to reveal biological mechanisms at the molecular level. In particular, differentially expressed proteins at different stages of daylily flower senescence have been detected using proteomics [23], while transcriptomics allowed the identification of 23 key genes related to flowering rhythm in *Hemerocallis* sp. [10]. Lipidomics based on analytical chemistry and statistical analysis have been used to comprehensively study the genes involved in lipid pathways and explain their physiological significance in lipid metabolism through large-scale analysis and quantitative studies [24]. In addition, lipidomics using liquid chromatography combined with tandem mass spectrometry (LC–MS/MS) has enabled the large-scale determination of lipid levels and their associated lipid molecular species [25]. Transcriptomics and lipidomics have also been combined to analyse the characteristics of glycerides during flower development in Arabidopsis [26]. However, few studies have combined transcriptomics and lipidomics to better understand glycerophospholipid biosynthesis and metabolism in daylilies.

Traditionally, research on daylily has only been focused on one or a few substances and genes at any given time point. Moreover, glycerophospholipid-associated signals and molecular events related to senescence during the flowering of daylilies remain unclear. In the present study, we have provided a comprehensive and dynamic overview of lipid metabolism in daylilies by combining lipidomics and transcriptomics to analyse the changes in the glycerophospholipid metabolism pathway during the flowering process. In addition, we identified characteristic genes and lipid categories to provide a basis for future studies on daylilies and their glycerophospholipid contents.

## Results

### Fluorescence localisation of membrane lipids in petals

**Fig. 1:**
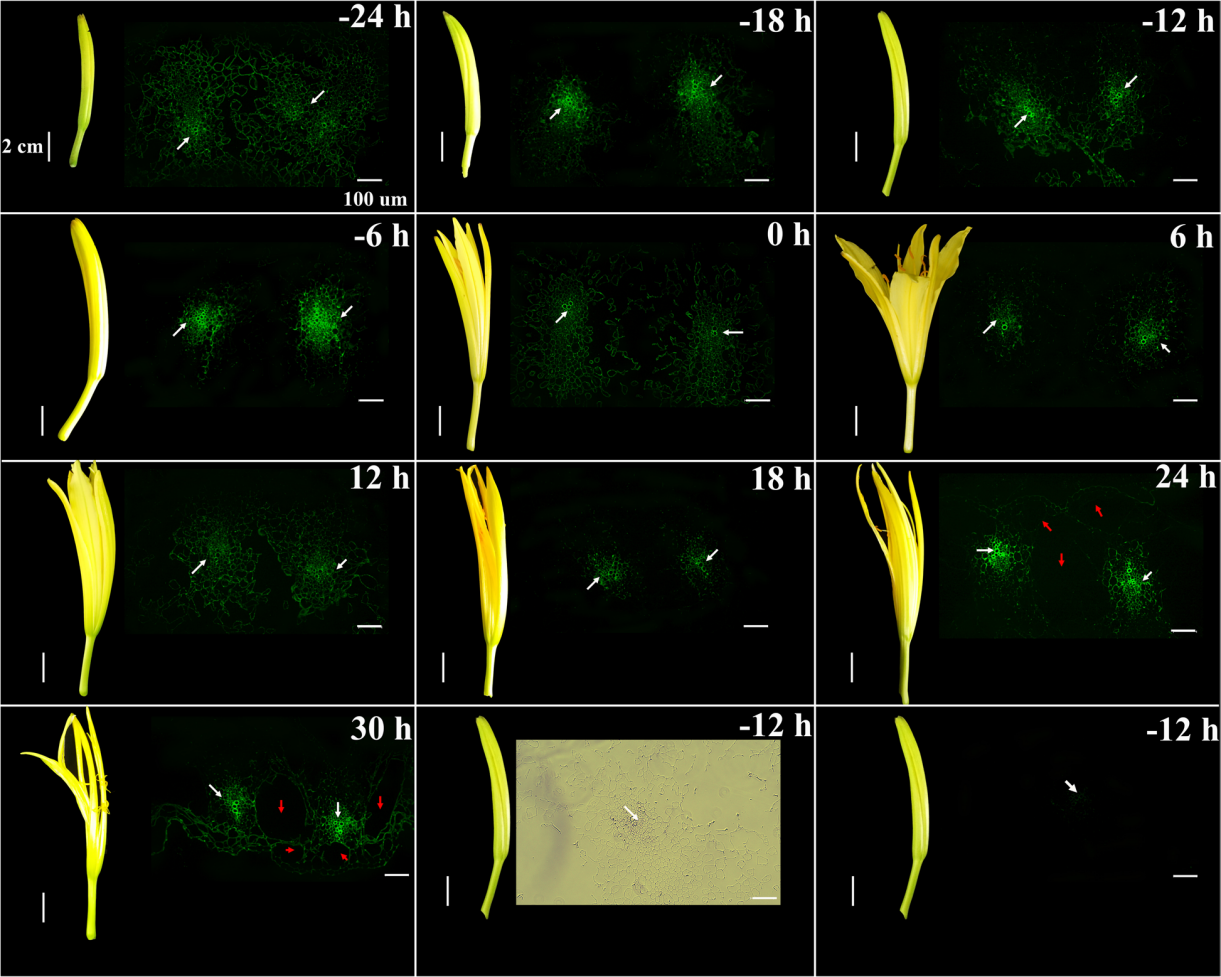
PKH67 fluorescent antibody labelling of daylily petal membrane lipids at different stages of flowering. (− 24 h to + 30 h) Fluorescence images of daylily petals 24 h before flowering to 30 h after flowering. The last two pictures are a bright-field image and a fluorescence image of a daylily petal 12 h before flowering without fluorescence labelling, respectively. White arrows indicate vascular bundles. Red arrows indicate petal tissue cavities. Scale bar = 2 cm for flower images. Scale bar = 100 μm for fluorescence images

First, we visualised the membrane lipids of daylily petals using PKH67 immunofluorescent antibodies (1:500; Sigma Aldrich, St. Louis, MI, USA). The tissue morphology and fluorescence levels of membrane lipids in the cells of the petals changed significantly during development from 24 h before flowering to 30 h after flowering (Fig. 1). The membrane lipids in the cells of the flower tissues were evenly distributed 24 h before flowering; however, the fluorescence intensity increased at 0 h when the tepals were slightly opened and was the highest around the vascular bundles in petals. When the petals opened, membrane lipid fluorescence decreased by varying degrees and became weaker around the vascular bundle, suggesting that membrane lipid levels were reduced to a certain extent. The gap between petal tissues was enlarged, and the intercellular space was evident at 24 h after flowering, indicating that the tissue had been damaged during senescence. As expected, almost no fluorescence was observed when the tissue was observed without immunofluorescence labelling 12 h before flowering (Fig. 1). However, the combination of bright field imaging and PKH67 immunofluorescence labelling allowed effective visualisation of petal membrane lipids. In summary, the bilayer membrane lipids were arranged orderly to perform effective functions before flower opening. In the later stage of flower opening, the molecular structure of membrane lipids was destroyed, tissue cavities appeared, and the membrane function was impaired.

### Membrane permeability, malondialdehyde (MDA) content, and membrane lipid-related enzyme activity

**Fig. 2:**
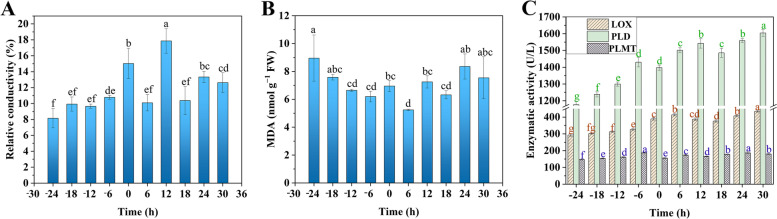
Relative conductivity (**A**), malondialdehyde (MDA) content (**B**), and membrane lipid-related enzyme activities (**C**) during daylily flowering. Different letters indicate significant differences (*p* < 0.05)

Next, we measured the relative conductivity and MDA content of daylily samples during flowering. Electrical conductivity, which indicates cell membrane permeability, increased at 0 h when the tepals were slightly open, peaked at 12 h after flowering (17.86%, *p* < 0.05), and then showed a non-significant decrease during wilting and senescence (Fig. 2A, B). Conversely, MDA content first decreased to 5.24 nmol g^−1^ fresh weight (FW) 6 h after flowering and then gradually increased. These results suggested that cell membrane lipids were intact and functioned normally from early bud development to tepal opening. Conversely, membrane lipids were damaged, cell permeability increased, and MDA content increased 6 h after flowering due to membrane lipid oxidation; however, the cells could still function normally and resist senescence. At 18 h after flowering, most cell functions were lost, and the increased permeability and membrane lipid peroxidation could not be reversed.

In addition, the activities of lipoxygenase (LOX), PLD, and phospholipid-N-methyltransferase (PLMT) were closely associated with lipid metabolism in the cell membrane (Fig. 2C). Notably, LOX and PLD activities tended to increase during daylily development and senescence, whereas PLMT activity first increased and then decreased. LOX activity was 413.3 U/L 6 h after flowering and then decreased from 18 to 24 h before peaking at 30 h (434.6 U/L). Conversely, PLD activity increased continuously until 6 h before flowering, then decreased slightly at 18 h after flowering, and peaked (1,603.9 U/L) at 30 h after flowering. The activity of both the enzymes decreased approximately 18 h after flowering, possibly due to cellular resistance to lipid degradation and increased enzyme activity during this period. The increase in enzyme activity 24 h after flowering indicated that cells were damaged, reducing their ability to resist senescence, while lipid oxidation increased enzyme activity. Since PLMT plays a key role in phospholipid synthesis during lipid metabolism, the peak in its activity during bud development at 6 h before flowering (186.7 U/L) suggests that phospholipid synthesis is active at this stage, generating large amounts of phospholipids to support membrane integrity and cell function. Collectively, these results demonstrated that various indicators were normal in the early stage of daylily flowering and the cell function was maintained. However, the membrane lipid function was impaired in the later stage of flowering and the cell function was inhibited to varying degrees.

### Lipidomic changes during flowering

**Fig. 3:**
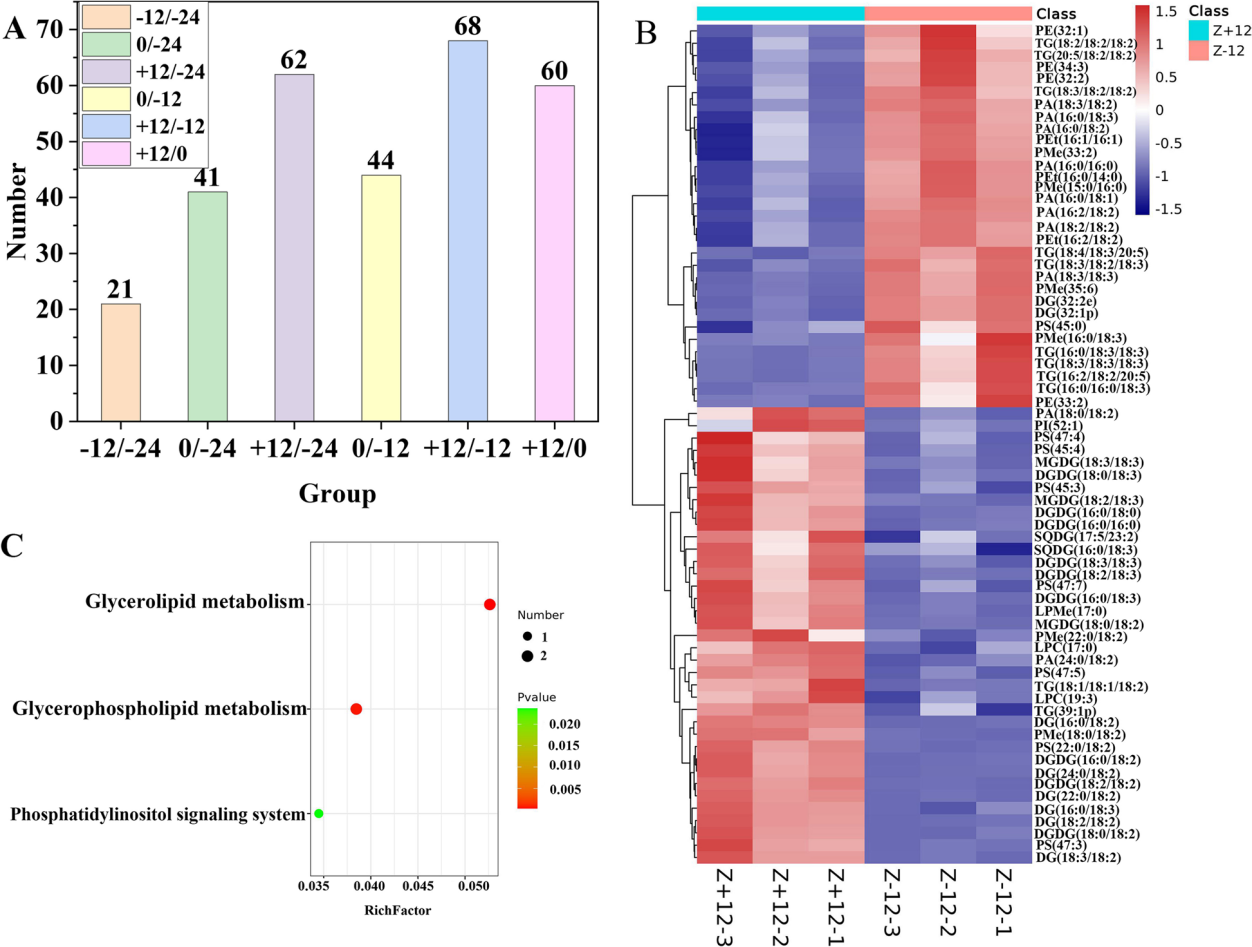
Lipidome differences and KEGG enrichment analysis. **A** Statistical analysis of lipid differences between each group. **B** Heatmap of the lipids that differed between the − 12 h and + 12 h groups. **C** KEGG pathway enrichment analysis of the different lipids

Generally, daylilies are used for food production 12 h before flowering (− 12 h). Considering the differences observed in lipid metabolites before and after flowering, we detected the lipid groups present in daylily samples at − 24, − 12, 0, and + 12 h of flowering. Lipidomic analysis revealed 558 lipids (Table S1), including 199 glycerolipids, 269 phospholipids, 39 sphingolipids, 50 glycolipids, and one sterol (Table 1). Notably, the greatest number of differences in lipids (68) were observed between − 12 h and + 12 h samples, followed by 62 lipid differences between − 24 h and + 12 h samples, and 21 lipid differences − 24 h and − 12 h samples (Fig. 3A, B). The most common lipid difference between all groups was triacylglycerol(18:3/18:2/18:3). The volcanic map showed that 37 differential lipids were significantly up-regulated and 31 were significantly down-regulated in the + 12 h/-12 h group (*P* < 0.05); however, 21 species were significantly up-regulated and 20 species were significantly down-regulated in the 0 h/-24 h group (Fig. S1). Kyoto Encyclopaedia of Genes and Genomes (KEGG) enrichment analysis revealed that the different metabolites in each group were enriched in the glycerolipid, glycerophospholipid, and phosphatidylinositol (PI) metabolic pathways (Fig. 3C). These results indicated that these three lipid metabolic pathways play an important role and that the lipid metabolism is highly dynamic and varies during the flowering of daylilies.

**Table 1:** Lipidomic analytes in daylilies

Lipids	Name	Abbreviation	Number	Total
Glycerolipids	Triacylglycerol	TG	149	199
	Diacylglycerol	DAG	48	
	Monoglyceride	MG	2	
Phospholipids	Phosphatidic acid	PA	44	269
	Phosphatidylcholine	PC	8	
	Phosphatidylethanolamine	PE	58	
	Phosphatidylglycerol	PG	36	
	Phosphatidylinositol	PI	13	
	Phosphatidylserine	PS	19	
	Lysophosphatidylcholine	LPC	18	
	Lysophosphatidyl ethanolamine	LPE	6	
	Lysophosphatidylglycerol	LPG	2	
	Lysophosphatidic acid	LPA	1	
	Cardiolipin	CL	2	
	Phosphatidylmethanol	PMe	36	
	Lyso dimethyl phosphatidyl ethanolamine	LdMePE	3	
	Lyso phosphatidylmethanol	LPMe	9	
	Phosphatidylinositol phosphate	PIP	3	
	DimethyI phosphatidylethanolamine	dMePE	1	
	Phosphatidylethanol	PEt	9	
	Lyso Phosphatidylethanol	LPEt	1	
Sphingolipids	Ceramide	Cer	17	39
	Glucosylsphingosine	SoG1	16	
	Coenzyme	Co	1	
	Sphingosine	So	4	
	Dihexosyl N acetylhexosyl ceramide	CerG2GNAc1	1	
Glycolipids	Monogalactosyl diglyceride	MGDG	12	50
	Digalactosyl diglyceride	DGDG	24	
	Sulfoquinovosyl diacylglycerol	SQDG	9	
	Monogalactosyl monoacylglycerol	MGMG	3	
	Digalactosyl monoacylglycerol	DGMG	2	
Sterols	Sitosterol ester	SiE	1	1

### Transcriptomic changes during flowering

**Fig. 4:**
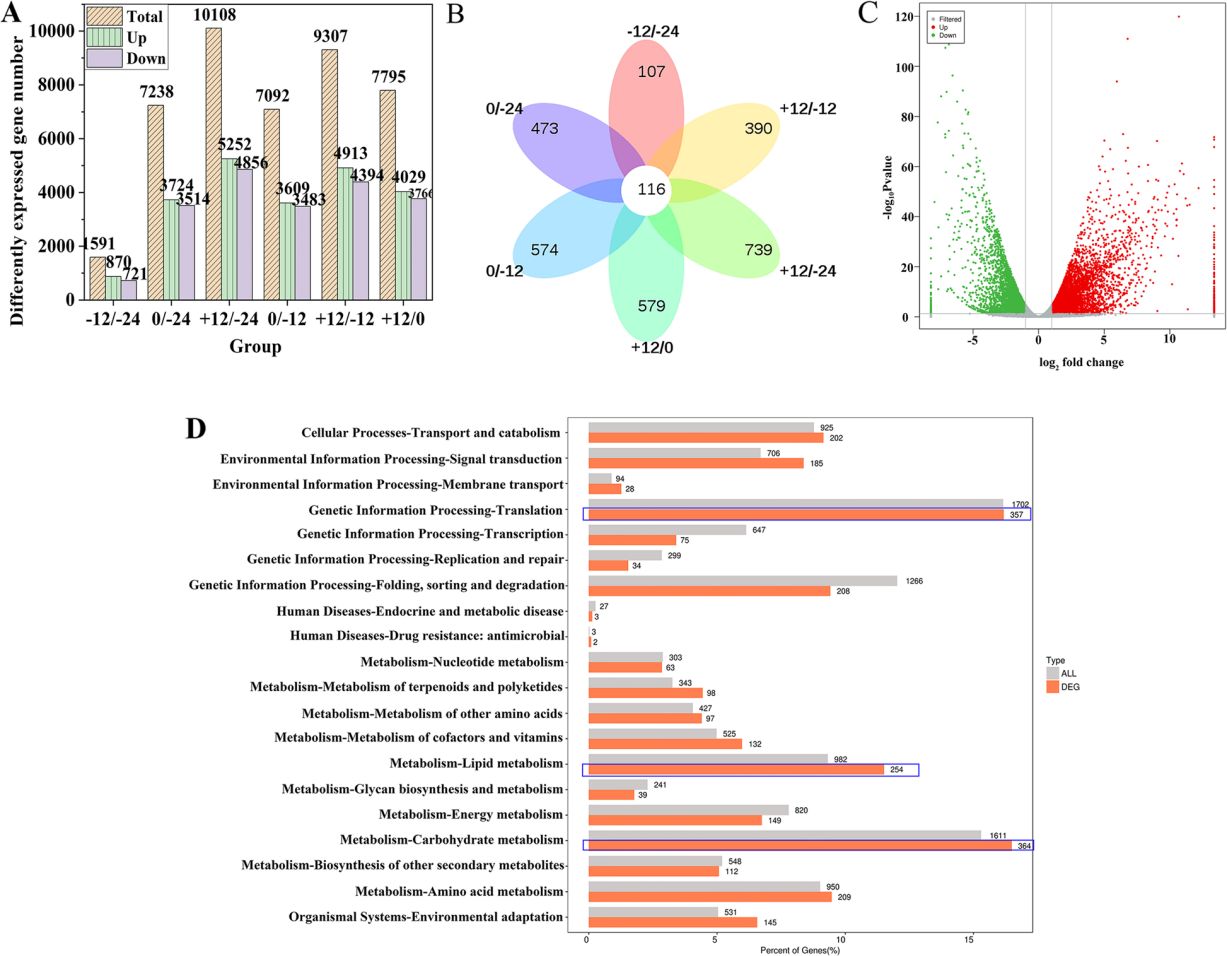
Transcriptome analysis of differentially expressed genes (DEGs) during daylily flowering. **A** DEGs in each comparison group. **B** Venn diagram of DEGs in each group. **C** Up (red)- and down (green)-regulated DEGs between the − 12 h/ + 12 h groups. Gray indicates no difference. **D** DEG distribution in metabolic pathways

A total of 80.51 Gb clean reads were detected in the daylily transcriptome at − 24, − 12, 0, and + 12 h of flowering, with an average of 6.05–7.04 Gb per sample, 93.9–94.43% Q30 bases, and 46.49% GC content. The reads from each sample were compared to the reference genome to evaluate the dynamic changes during daylily flowering, with an alignment rate of 91.77–92.13%. Subsequent protein-coding gene expression analysis revealed six groups containing 1591, 7238, 10,108, 7092, 9307, and 7795 differentially expressed genes (DEGs) (Fig. 4A). The number of common differential genes in the comparison group which combined by -24, -12, 0 and 12 h were 107 (− 12/ − 24), 390 (+ 12/ − 12), 739 (+ 12/ − 24), 579 (+ 12/0), 574 (0/ − 12), 473 (0/ − 24) respectively, 116 of which were common among the six groups (Fig. 4B). The comparison between the − 24 h and + 12 h groups revealed the greatest number of DEGs, with 5252 upregulated and 4856 downregulated genes (Fig. 4A); the volcanic map is shown in Fig. 4C. KEGG enrichment analysis revealed that most DEGs were related to carbohydrate metabolism, followed by genetic information processing-translation, and lipid metabolism (Fig. 4D). These results suggested strong transcriptional changes during daylily flowering, with lipid metabolism playing an important role.

Next, we screened all DEGs involved in glycerophospholipid metabolism to analyse their significance in each group. Twenty-two common DEGs were found between the two 24 h groups (Table S2) that were divided into 13 categories: glycerol-3-phosphate dehydrogenase 1, *PLMT*, diacylglycerol kinase (*DGK*), phospholipase A (*PLA*), phosphoethanolamine N-methyltransferase (*PEAMT*), *PLD*, lysophospholipase A, glycerol-3-phosphate acyltransferase, lysophospholipid acyltransferase, choline kinase (*CK*), triacylglycerol lipase SDP1-like, diacylglycerol diphosphate phosphatase (*DPP*), and glycerophosphodiester phosphodiesterase (*GDE*). The enzymes encoded by these genes play important roles in glycerophospholipid metabolism: PLMT, PEAMT, and CK are rate-limiting enzymes involved in lecithin synthesis, while PLD and DGK can degrade phospholipids to produce signal molecules such as phosphatidic acid (PA) and diacylglycerol (DAG). Six of these screened genes were detected using quantitative real-time PCR (qRT-PCR), and their relative expression was consistent with the results of transcriptome analysis, confirming the reliability of the transcriptome data (Fig. S2). These data indicated that glycerophospholipid metabolism is regulated by many genes at different levels during daylily flowering.

### Combined analysis of the glycerophospholipid metabolism pathway

**Fig. 5:**
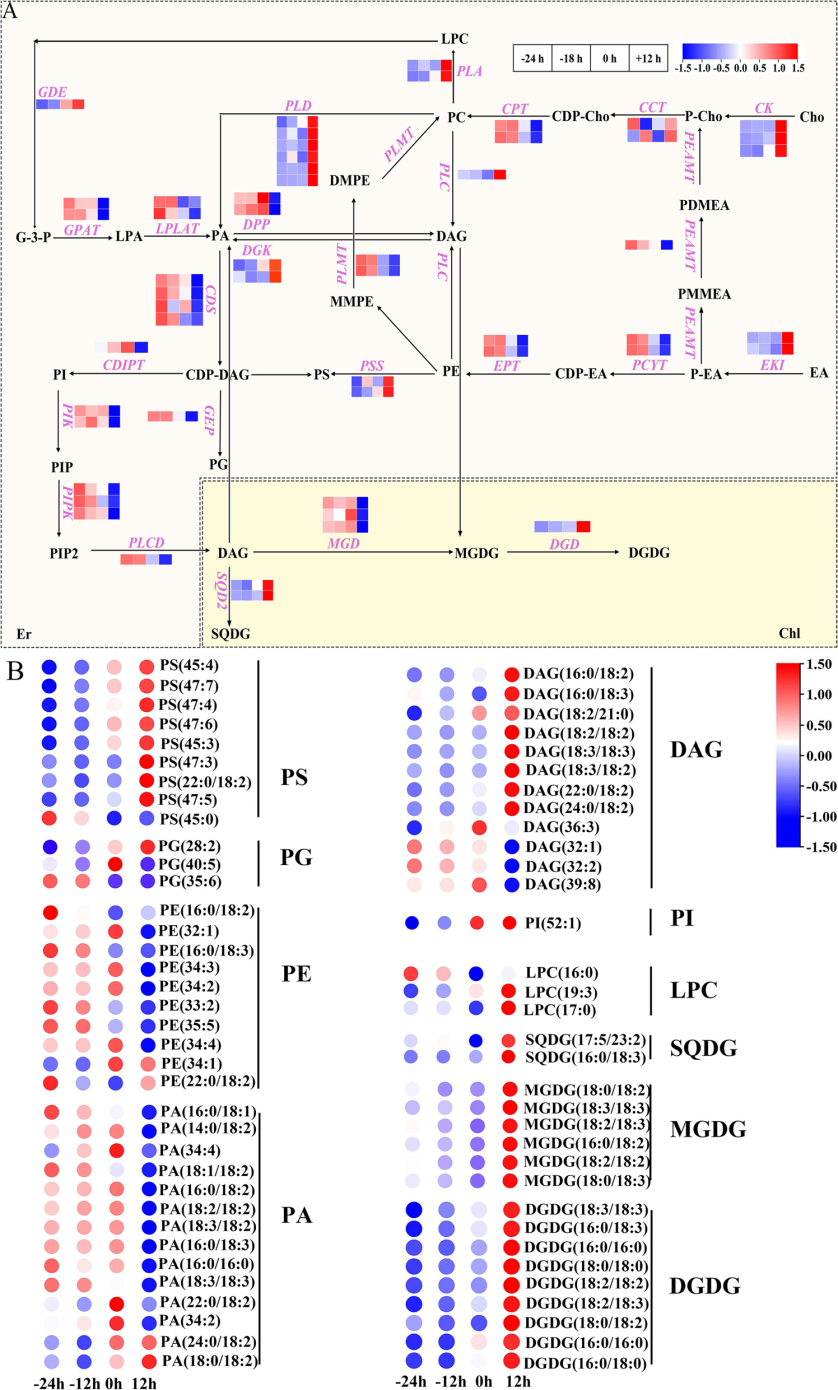
Genes and lipids in glycerophospholipid metabolism. **A** DEG expression in the glycerophospholipid metabolism pathway (Refer to KEGG pathway database). **B** Differential lipid expression in the glycerophospholipid metabolism pathway

As the transcriptome analysis revealed that the DEGs screened during daylily flowering played important roles in the glycerophospholipid metabolic pathway, we next identified DEGs of the same type in the transcriptome with a correlation coefficient > 0.8 and plotted the glycerophospholipid metabolic pathway (Fig. 5A). Simultaneously, we screened different lipids in the glycerophospholipid metabolic pathway using lipidomics. The combined analysis of these two sets of data revealed that ethanolamine kinase genes (*EKI*) in the phosphatidylethanolamine (PE) synthesis pathway were initially upregulated during daylily flowering; however, the key genes coding for ethanolamine phosphotransferase (*EPT*) and ethanolamine-phosphate cytidylyltransferase (*PCYT*) were downregulated, leading to a significant decrease in PE(16:0/18:2), PE(32:1), PE(16:0/18:3), PE(34:3), PE(32:2), PE(33:2), PE(34:4), and PE(35:5) levels. In the PC pathway, the initial synthesis genes were upregulated; however, in the consecutive methylation pathway, both *PEAMT* and its key synthesis genes diacylglycerol cholinephosphotransferase (*CPT*) and *PLMT* were downregulated, resulting in no significant difference in PC(16:0/18:3), PC(34:2), PC(34:3), PC(36:6), PC(36:2), PC(36:4), PC(34:1), and PC(36:5) levels (Table S1). Furthermore, the expression of lysophosphatidylcholine (LPC)(19:3) and LPC(17:0), a hydrolysate of phosphatidylcholine, increased due to the degradation of the latter following *PLA* upregulation, while phosphatidylserine (PS) levels were increased by upregulated phosphatidylserine synthase (Fig. 5B). Thus, highly dynamic changes in glycerophospholipids, which are the main components of the cell membrane, were observed during flowering.

Due to the upregulation of *PLD* and phospholipase C (*PLC*) in the glycerophospholipid pathway, the glycerophospholipid hydrolysates PA and DAG, which are important signalling molecules in plants, were temporarily upregulated. However, transcriptome and lipidome analyses based on the complex network of synthesis and degradation pathways showed that the signalling molecules displayed a downward trend during the flowering process. In addition, phosphatidylinositol kinase gene, phosphatidylinositol phosphate kinase gene, and *PLC/D* were sequentially downregulated in the dual messenger signalling pathway, which could cause the decrease in DAG(36:3), DAG(32:1), DAG(32:2), and DAG(39:8) levels observed in the lipidome analysis. Collectively, these results indicated that signal transduction is gradually downregulated during the later stages of daylily flowering alongside the weakening of cell structure and tissue function. Consistently, digalactosyl diglyceride (DGDG) and sulfoquinovosyl diacylglycerol (SQDG), which play important roles in the chloroplast membrane, were upregulated due to the increased DGDG synthase (*DGD*) and SQDG synthase (*SQD*) expression.

**Fig. 6:**
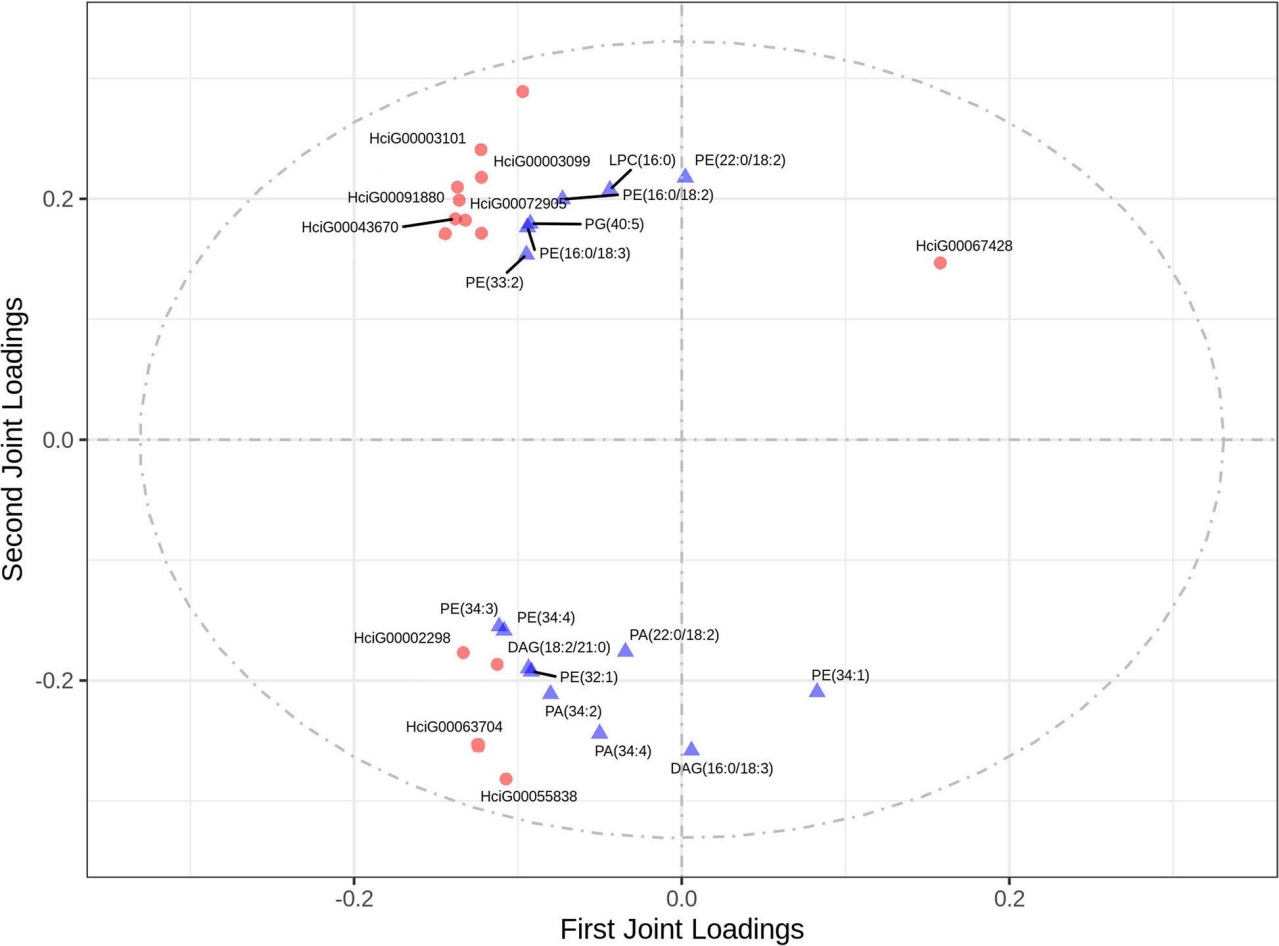
O2PLS loadings plot of metabolites and transcripts involved in daylily flowering. Circles and triangles represent individual transcript and metabolite loading values, respectively

To comprehensively explore glycerophospholipid metabolism during daylily flowering, we used two-way orthogonal partial least squares (O2PLS) for combined transcriptome and lipidomic analysis (Fig. 6). A total of 52 transcripts and 69 (Table S4) metabolites in the glycerophospholipid pathway were used to construct the model (R^2^X = 0.944, R^2^Y = 0.957). The 15 most closely related genes and lipids are shown in Fig. 6. *DPP* (HciG00002298 and HciG00063704) was strongly and positively correlated with PA(22:0/18:2), PA(34:2), PA(34:4), and DAG(18:2/21:0), whereas these lipid signalling molecules were strongly and negatively correlated with *PLC* (HciG00067428). In addition, *EPT* (HciG00043670), ethanolamine-phosphate cytidylyltransferase (HciG00072905), and *PLMT* (HciG0003099, HciG0003101) were positively correlated with PE(16:0/18:2), PE(16:0/18:3), PE(33:2), and LPC 16:0) but negatively correlated with PE(34:1). Collectively, these results indicated that different genes regulate different substances during the flowering process and that their synthesis and catabolic pathways form a highly complex network.

## Discussion

Lipids are important structural and functional membrane components that play key roles in maintaining cell homeostasis, membrane transport, and signal transduction [27]. To date, few studies have been reported on glycerophospholipid metabolism and phosphatidylinositol signalling pathway in plants such as *Arabidopsis thaliana* [26], soybean [28], blueberry [29], and bell peppers [30], tobacco [31]. However, the lipids in daylilies, particularly the dynamic changes in glycerophospholipid and lipid signalling molecules during the flowering process, have not yet been reported. Glycerophospholipids constitute one of the eight lipid groups and include PE, PC, phosphatidylglycerol (PG), PI, PS, PA, and lysophospholipids [32]. The structure, function, composition, and synthesis pathways of glycerophospholipids vary dramatically during cell division, differentiation, and senescence. PE synthesis proceeds via two pathways: the PS decarboxylation pathway, which accounts for a small proportion of PE synthesis in plants, and the CDP-ethanolamine pathway, which is the main source of PE in plants and whose rate-limiting enzyme is EPT [33]. The *Arabidopsis thaliana* genome encodes two EPT (or AAPT) genes with dual specificity. Plants with mutations in the *aapt1* or *aapt2* genes show no-growth phenotypes, while double knockout mutants that are hemizygous for either *aapt1* or *aapt2* display impaired pollen and seed development, leading to embryonic lethality of the double knockout plants [34]. PC synthesis involves two primary pathways: the CDP-choline pathway and the triple successive methylation pathway [35]. The rate-limiting enzyme of the latter is PEAMT [36]. CDP-diacylglycerol is a hydrolysate of PE and PC that can be used to synthesise PG and PI, which are also substrates for PS synthesis [33]. As lipid synthesis and degradation are regulated through a large and complex cellular network, further studies are required to fully elucidate the underlying regulatory mechanisms.

Senescence and adverse damage in plant cells are closely related to membrane lipid peroxidation induced by the accumulation of reactive oxygen species. As MDA is an important product of membrane lipid peroxidation, it can be a useful indicator of the degree of membrane lipid peroxidation and membrane damage [37]. Cell membranes generally exhibit selective permeability; however, when plants undergo aging or are affected by the environment, the electrolytes in the cells experience osmosis, causing an increase in electrical conductivity and ion leakage due to changes in membrane structure and lipid composition [29, 38]. Four lipid-degrading enzymes, PLD, PA phosphatase, lipolysis acylhydrolase, and LOX, are closely associated with plant senescence [39]. Therefore, in the present study, we measured the relative conductivity, MDA content, and PLD and LOX activities, which are closely related to cell membrane characteristics, to detect changes in the cell membrane during daylily flowering. Interestingly, a normal physiological function was detected in daylily cells during the early stages of flowering; however, the cell membrane appeared damaged and cell function was affected during the later stages. Previous studies have demonstrated that PLD activity is positively correlated with the rate of senescence in castor bean leaves and could trigger the calcium signalling pathway to regulate additional cell processes [15]. LOX catalyses lipid oxidation, which produces superoxide radicals capable of inducing lipid peroxidation [39]. In addition, the metabolism of membrane lipids, especially phospholipids, can reduce bilayer volume lipid saturation, thereby affecting membrane conformation, boundary defects, content leakage, and ultimately reducing function [39]. Our findings, together with those of previous studies, indicate that the membrane lipid-related properties of daylilies change dynamically and substantially before and after flowering. Therefore, it is necessary to study the specific lipids and genes that underlie these changes in daylilies.

Unfortunately, studying the changes in membrane lipids in detail [40] is challenging due to the complexity of the membrane lipid molecules and limitations of the available analytical methods. Here, we used both transcriptomics and lipidomics to identify changes in the genes and lipids during daylily flowering. The findings are consistent with the notion that gene expression precedes material accumulation.

Cell membrane glycerophospholipids are a potent source of synthetic lipid messengers, which are characterised by transient accumulation and rapid turnover [31]. Phospholipases (PLs) are key enzymes that catalyse the initiation of lipid hydrolysis and produce signalling molecules [41]. The three main PLs in the glycerophospholipid pathway are PLD, PLC, and PLA. The results of our study suggested that glycerophospholipid hydrolysis was increased during the later stages of flowering.

PA synthesis and decomposition occur via multiple pathways and are therefore highly dynamic and complex [42]. PA promotes the negative curvature of the membrane bilayer and increases the permeability of membrane vesicles in vitro [43]. Furthermore, high PA levels can disrupt membrane stability, thereby causing the loss of membrane integrity and membrane protein function during injury, senescence, freezing, and drought [42]. In this study, the lipidome analysis showed that PA levels decreased during flowering, whereas DAG, monogalactosyl diglyceride (MGDG), and DGDG were upregulated, likely due to PA dephosphorylation by PA phosphatase to generate DAG, which was converted into MGDG by MGDG synthase [26]. MGDG and DGDGs are unique and abundant structural glycolipids [40] in photosynthetic membranes with the delicate framework of the plasma membrane [44]. Here, transcriptome and lipidome analyses revealed that *DGD* and *SQD* were upregulated during flowering while the MGDG, DGDG, and SQDG content was increased, which stabilised the photosynthetic system II complex [29]. Unlike that observed for cytoplasmic membranes, senescence does not significantly change the phase transformation or lipid composition of the thylakoid membranes of chloroplasts [39], which may explain why the MGDG, DGDG, and SQDG levels increased in the present study.

Paul Karl Horan (PKH) lipophilic, long-chain carbocyanine dyes are used to label artificial membranes and biofilms. The aliphatic tails of PKH dyes, such as PKH26 and PKH67, are rapidly inserted into the exposed lipid bilayer, resulting in the formation of strong non-covalent interactions that promote long-term retention and stable fluorescence while generally not affecting cell growth, viability, or proliferation. Consequently, PKH dyes have been widely used to label cells for tracking both in vivo and in vitro [45, 46]; however, PKH dyes have rarely been applied to plant materials. In the present study, we used PKH67 to determine the fluorescence localisation of lipids in daylily petals for the first time. The images of PKH fluorescence staining are very clear, indicating that this method is feasible in the study of daylily membrane lipids.

## Conclusions

To determine the fluorescence localisation of lipids, we used PKH67 in daylily petals for the first time. The resulting images indicated that membrane lipids were extensively damaged in the later stages of flowering and affected the membrane, internal structure, and functions of cells. Furthermore, the results showed that MDA content and relative conductivity, which are closely related to membrane lipids, decreased after 18 h of flowering and that the cellular changes were difficult to reverse. Conversely, the activity of PLD and LOX increased during daylily flowering, indicating that hydrolysis and lipid oxidation intensified, which may lead to changes in membrane lipid conformation and membrane damage. Thirteen DEGs related to glycerophospholipids were identified in the daylily samples and played important roles in glycerophospholipid pathways while significantly regulating lipids. Daylily flowering is mainly regulated via the DAG and PA signalling pathways through PC and PE hydrolysis as well as the PI signalling pathway. *DPP* expression was significantly and positively correlated with the contents of PA(22:0/18:2), PA(34:2), PA(34:4), and DAG (18:2/21:0); however, *PLC* expression was negatively correlated with their content. *EPT* and *PLMT* expression were significantly and positively correlated with the contents of PE(16:0/18:2), PE(16:0/18:3), PE(33:2), and LPC(16:0), but negatively correlated with that of PE(34:1). This study provides novel insights into the glycerophospholipid metabolism of daylilies.

## Materials and methods

### Materials

‘Datong Huanghua 6’ plants were cultivated in the *Hemerocallis* germplasm nursery at Shanxi Agricultural University (Taigu, China). Daylily buds were sampled 24, 18, 12, and 6 h before flowering ( −), at tepal bloom (0 h), and 6, 12, 18, 24, and 30 h after flowering ( +). A total of 10 samples were collected from − 24 to + 30 h. Samples for transcriptome and lipidome analyses were collected at − 24, − 12, 0, and + 12 h.

### Immunofluorescence labelling of membrane lipids

Daylily petal samples collected at different time points were cut into 0.5 cm pieces and fixed in 38% formaldehyde: glacial acetic acid: 70% ethanol fixative (1:1:18, v/v) for over 24 h before embedding them in paraffin. The tissue sections were then dried in an oven at 65 ℃ for 2 h, placed in xylene for 10 min, dewaxed twice, and hydrated in an ethanol series (100%, 95%, and 80%) with purified water for 5 min each [47, 48]. After incubation in 0.5% Triton X-100 [prepared in phosphate-buffered saline (PBS)] for 20 min at 25 ℃, the slides were immersed in PBS (pH 7.4) three times for 5 min each and then sealed with 5% bovine serum albumin at 37 ℃ for 30 min. Once the sealing fluid around the tissues had been aspirated, the slides were incubated in diluted membrane lipid fluorescent antibody PKH67 (1:500, Sigma Aldrich) in a humid box at 37 °C for 1.5 h and then sealed with a plate sealing solution (YZB, BaSO) containing an anti-fluorescence quenching agent [49, 50]. The slides were imaged using a fluorescence microscope (CKX53; Olympus, Tokyo, Japan; 4 × 100).

### Determination of membrane permeability

The daylily samples were washed separately, rinsed with deionised water, and punched into 30 discs per group after the surface moisture had dried. The discs were divided into three clean test tubes (*n* = 10 per tube) containing 15 mL of deionised water. The tubes were sealed with plastic wrap, which was punctured with an anatomical needle, and then air pumped for 10 min to create vacuum. The intake valve was then opened slowly to allow the petals to sink. After 30 min, the initial conductivity was measured using a conductometer (5430R; Eppendorf, Hamburg, Germany) with deionised water as a blank control group. After the tubes had been incubated in boiling water for 10 min and cooled to 25 ℃, the final conductivity was measured. Membrane permeability was calculated as the relative conductivity (%) [29].

### Determination of MDA content

The daylily samples (1 g) were added to 5 mL of 10% (w/v) trichloroacetic acid in an ice bath, ground into a homogenate, and centrifuged (10,000 rpm) at 4 ℃ for 20 min. Next, 2 mL of supernatant was added to 2 mL of 0.67% (w/v) thiobarbituric acid, boiled for 20 min, and cooled to 25 ℃. After further centrifugation (10,000 rpm), the absorbance of the supernatant was detected at 450, 532, and 600 nm (UV5200, Shanghai Metash Instruments Co., Ltd., China). Distilled water was used as the control. MDA content was estimated using the following formula: MDA (nmol g^−1^ FW) = [6.45*(A532 – A600) – 0.56*A450]*5 [29].

### Determination of membrane lipid-related enzyme activity

Enzyme-linked immunosorbent assays (ELISAs) were used to detect the activities of LOX, PLD, and PLMT, all of which are related to membrane lipid metabolism. Briefly, 0.1 g of daylily was ground in liquid nitrogen, extracted using 1 mL 80% methanol, and incubated at − 20 °C for 12 h. After centrifugation at 8000 rpm and 4 °C for 1 h, the supernatant was collected and purified using a C-18 solid-phase extraction column balanced with 80% methanol (1 mL). The eluent was successively eluted with 100% methanol (5 mL), 100% ether (5 mL), and 100% methanol (5 mL). After the samples had been vacuum-dried, they were incubated in PBS buffer (1 mL total volume; pH 7.4) at 25 ℃ for 30 min, followed by centrifugation at 8000 rpm and 4 ℃ for 15 min. The supernatant was collected to perform ELISAs according to the manufacturer’s instructions (Shanghai Enzyme-linked Biotechnology, Shanghai, China) [30].

### Lipidome analysis

First, 60 mg of each daylily bud was incubated at − 20 ℃ for 2 min with 20 μL 0.01 mg/mL internal standard Lyso PC17:0 (methanol solvent) and 300 μL methanol–water (1:1, v/v), vortexed for 30 s, and crushed using a high-throughput tissue crusher for 2 min at 60 Hz. The samples were then vortexed with 300 μL chloroform for 30 s, extracted using sonication in an ice-water bath for 10 min, and incubated at − 20 °C for 20 min. After centrifugation at 12,000 rpm and 4 °C for 10 min, the lower chloroform layer (200 μL) was pipetted into an LC–MS injection vial. Chloroform–methanol (300 μL; 2:1, v/v) was added to the remaining solution; the mixture was vortexed for 30 s and extracted using ultrasonication for 10 min in an ice-water bath. The extracted samples were left to stand for 20 min at − 20 °C and then centrifuged (12,000 rpm); thereafter, 300 μL of the lower chloroform layer was added to the previous LC–MS vial. The pooled sample was then dried in a vacuum desiccator. Thereafter, the samples were reconstituted in 200 μL isopropanol-methanol (1:1, v/v), vortexed for 30 s, sonicated for 3 min, and transferred to a 1.5 mL centrifuge tube which was left at − 20 °C for 2 h. After centrifugation (12,000 rpm), the supernatant (150 μL) was collected for LC–MS/MS analysis [51]. Quality control samples were prepared by mixing all sample extracts in equal volumes to the same total volume for each sample.

LC–MS/MS analysis was performed using a Dionex U3000 ultra-high performance liquid chromatography (UHPLC) system (Thermo Fisher Scientific, Waltham, MA, USA), equipped with an ACQUITY UPLC BEH C18 column (100*2.1 mm, 1.7 μm; Waters, Milford, MA, USA) at 60 ℃. Mobile phase A was acetonitrile: water (60:40, v/v), mobile phase B was acetonitrile: isopropanol (10:90, v/v), and both mobile phases contained 10 mmol/L ammonium formate. The injection volume was 5 μL, and the flow rate was 0.4 mL/min. MS was performed using positive and negative heated electrospray ionisation (HESI) modes in a Q Exactive instrument (Thermo Fisher Scientific). The HESI source conditions for positive ionisation mode were as follows: heater temperature, 350 °C; sheath gas flow rate, 50 arbitrary units (au); auxiliary gas flow rate, 15 au; sweep gas flow rate, 1 au; spray voltage, 3.8 kV; capillary temperature, 320 °C; S-Lens RF level 75%; MS_1_ scanning range, *m/z* 135–2000. For negative ionisation mode, all parameters except spray voltage (3.0 kV) were the same as in the positive ionisation mode. The mass charge ratio (*m/z*) of lipid molecules and lipid fragments was obtained using the following method: ten fragment profiles (MS_2_ Scan, HCD) were obtained after each full scan. MS_1_ had a resolution of 70,000 at *m/z* 200, and MS_2_ had a resolution of 17,500 at *m/z* 200. The raw data exported by the Q Exactive LC–MS/MS was read using LipidSearch software (Thermo Fisher Scientific), and the exact mass numbers of MSn and parent ions were read. Based on the parent ion and multistage MS data of each independent sample, the lipid molecular structure, and the addition mode of positive and negative ions were identified. The search results of each independent sample were aligned according to a certain retention time range, and the results were combined into a single report to sort out the original data matrix [52, 53].

### Transcriptome analysis

Total RNA was extracted from the − 24, − 12, 0, and + 12 h daylily samples using the mirVana miRNA Isolation Kit (Ambion, Austin, TX, USA) and stored at − 70 ℃. RNA concentration and quality were detected using a NanoDrop ND 2000 spectrophotometer (NanoDrop Technologies, Wilmington, DE, USA) and an Agilent Bioanalyzer 2100 System (Agilent Technologies, Palo Alto, CA, USA), respectively. A cDNA library was constructed using a TruSeq Stranded mRNA LTSample Prep Kit (Illumina, San Diego, CA, USA). Briefly, mRNA was used as a template to synthesise single-stranded cDNA, which was converted into purified double-stranded cDNA using a two-strand synthesis reaction system. After end repair, 3' A tail addition, and sequencing adapter addition, the templates were amplified using PCR. Following quality inspection, 150 bp paired-end reads were generated using an Illumina HiSeq™ 2500 system. The raw reads generated by high-throughput sequencing were subjected to quality control and linker removal. The remaining high-quality clean reads were compared to the daylily reference genome and annotated for proteins. The fragments per kilobase of transcript per million mapped reads of each gene was calculated and used to quantify the expression levels of the annotated genes. The nbinomTest function was used to calculate the *p* and fold-change values for the different comparisons. DEGs with *p* < 0.05 and 2 < fold change < 0.5 were subjected to gene ontology and KEGG enrichment analyses to determine their main functions and metabolic pathways, respectively [54, 55]. Lipidomic and transcriptomic profiles of glycerophospholipid metabolism pathway referenced the KEGG pathway database [56].

### qRT-PCR validation

Total RNA was extracted from each sample using a TaKaRa MiniBEST Plant RNA Extraction Kit (Takara, Dalian, China). RNA concentration and purity were detected using a NanoDrop ND 2000 (NanoDrop Technologies) spectrophotometer. cDNA was synthesised in an ice box using a Reverse Transcriptase Kit (PrimeScript™ RT, Takara, Dalian, China) according to the manufacturer’s instructions. All primers were designed using Primer Premier 5.0. Relative gene expression levels were calculated from the melting curve fluorescence signals using the 2^−ΔΔCt^ method, with AP4 as the reference gene [57].

### Statistical analyses

All experiments were repeated three times. Statistical analyses were performed using Excel 2019. SAS 8E was used to analyse significance, and Origin2019 and TBtools were used for mapping. The O2PLS analysis was performed to integrate metabolome and transcriptome data online (https://www.omicshare.com/tools). One-way analysis of variance with Duncan's test was conducted to evaluate significant differences between the means of multiple groups (*p* < 0.05).

## Supplementary Information

**Additional file 1: Table S1.** The lipids of lipidomic.

**Additional file 2: Figure S1.** Lipidomic differences in daylily.

**Additional file 3: Table S2.** Summary of key genes involved in the glycerophospholipid metabolism pathways. 

**Additional file 4: Figure S2.** Verification of key genes using qRT-PCR.

**Additional file 5: Table S3.** Primers of qRT-PCR.

**Additional file 6: Table S4.** The classes of lipids in lipidomic.

## Data Availability

The lipidomics datasets generated and analysed during the current study are available in the metabolights repository, with the website: www.ebi.ac.uk/metabolights/MTBLS5135.

## Abbreviations

CDP-choline: Cytidine-diphospho-choline
CK: Choline kinase
DAG: Diacylglycerol
DEGs: Differentially expressed genes
DGD: DGDG synthase
DGDG: Digalactosyl diglyceride
DGK: Diacylglycerol kinase
DPP: Diacylglycerol diphosphate phosphatase
ELISAs: Enzyme-linked immunosorbent assays
EPT: Ethanolamine phosphotransferase
FW: Fresh weight
HESI: Heated electrospray ionisation
KEGG: Kyoto Encyclopaedia of Genes and Genomes
LC–MS/MS: Liquid chromatography combined with tandem mass spectrometry
LOX: Lipoxygenase
LPC: Lysophosphatidylcholine
MDA: Malondialdehyde
MGDG: Monogalactosyl diglyceride
O2PLS: Two-way orthogonal partial least squares
PA: Phosphatidic acid
PBS: Phosphate-buffered saline
PC: Phosphatidylcholine
PE: Phosphatidylethanolamine
PEAMT: Phosphoethanolamine N-methyltransferase
PG: Phosphatidylglycerol
PI: Phosphatidylinositol
PKH: Paul Karl Horan
PLA: Phospholipase A
PLC: Phospholipase C
PLD: Phospholipase D
PLMT: Phospholipid-N-methyltransferase
PLs: Phospholipases
PS: Phosphatidylserine
qRT-PCR: Quantitative real-time PCR
SQD: SQDG synthase
SQDG: Sulfoquinovosyl diacylglycerol
UHPLC: Ultra-high performance liquid chromatography
