# Supplementary material for: Lipidomic and transcriptomic profiles of glycerophospholipid metabolism during Hemerocallis citrina Baroni flowering
Source: BMC Plant Biol. 2023 Jan 23;23:50. doi: 10.1186/s12870-022-04020-x (PMC9869519; doi:10.1186/s12870-022-04020-x)
Supplement: Supplementary file 2 — Additional file 2: Figure S1. Lipidomic differences in daylily. [file 12870_2022_4020_MOESM2_ESM.docx]

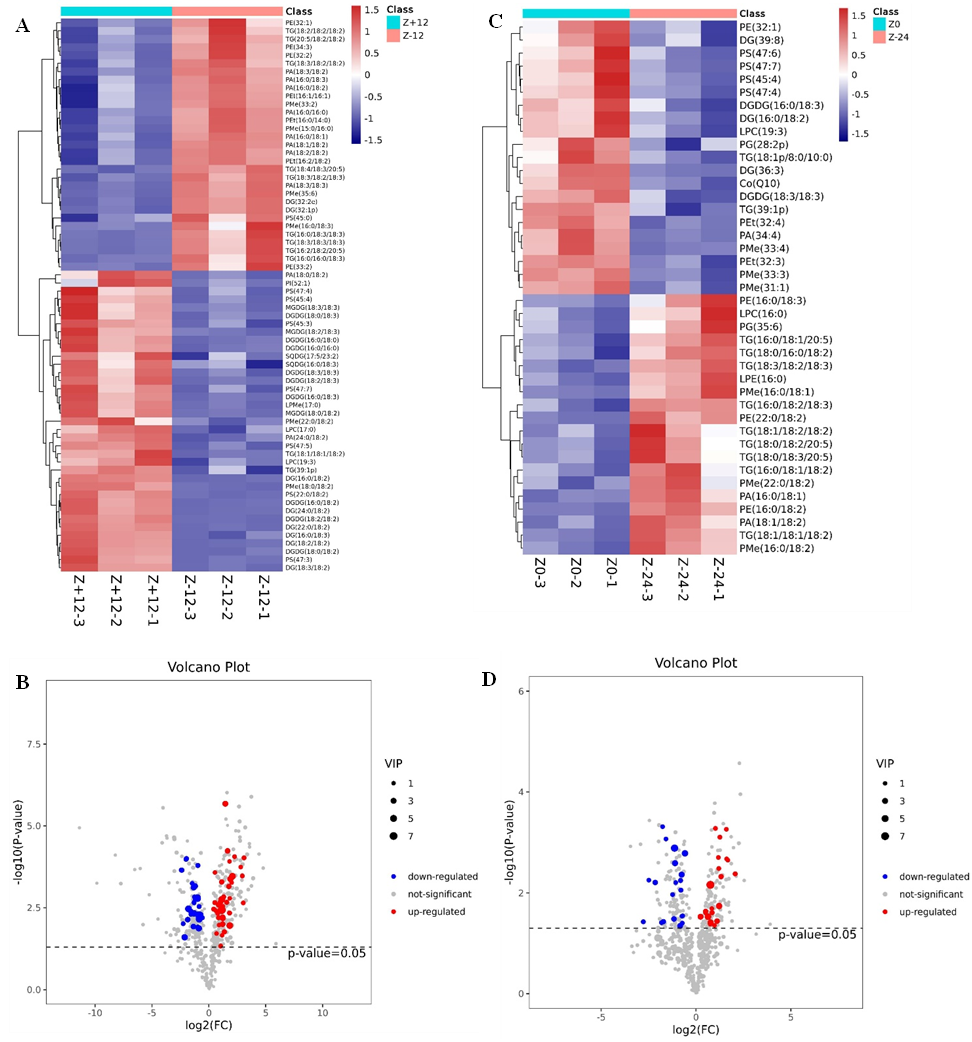
**Figure S1** Lipidomic differences in daylily. A and B are the heat map and volcano map between +12 h/-12 h, respectively, showing differential lipids; C and D are the heat map and volcano map between 0 h/-24 h, respectively, showing differential lipids. In the heat map, the abscissa represents the sample and the ordinate represents the differential lipids. The color from blue to red indicates the expression abundance of lipids from low to high, that is, the darker the red, the higher the expression abundance. In the volcano map, red points represents significantly up-regulated lipids, blue points represents significantly down-regulated lipids, and gray points represent non-significant results.
