# Supplementary material for: Lipidomic and transcriptomic profiles of glycerophospholipid metabolism during Hemerocallis citrina Baroni flowering
Source: BMC Plant Biol. 2023 Jan 23;23:50. doi: 10.1186/s12870-022-04020-x (PMC9869519; doi:10.1186/s12870-022-04020-x)
Supplement: Supplementary file 3 — Additional file 3: Table S2. Summary of key genes involved in the glycerophospholipid metabolism pathways. [file 12870_2022_4020_MOESM3_ESM.docx]

**Table S2** Summary of key genes involved in the glycerophospholipid metabolism pathways.

| Gene ID | Gene symbol | Description |
| --- | --- | --- |
| HciG00035194 | *GPD1* | glycerol-3-phosphate dehydrogenase |
| HciG00003099 | *PLMT* | phosphatidyl-N-methylethanolamine N-methyltransferase |
| HciG00003101 |  |  |
| HciG00055780 | *DGK* | diacylglycerol kinase |
| HciG00052992 | *PLA* | phospholipase A1 |
| HciG00014265 |  | phospholipase A2 |
| HciG00023306 | *PLD* | phospholipase D |
| HciG00092866 |  |  |
| HciG00020810 |  |  |
| HciG00064092 |  |  |
| HciG00047999 |  |  |
| HciG00091880 | *PEAMT* | phosphoethanolamine N-methyltransferase |
| HciG00072202 | *LYPLA* | lysophospholipase III |
| HciG00087088 |  | lysophospholipase II |
| HciG00022532 | *GPAT* | glycerol-3-phosphate acyltransferase |
| HciG00062365 |  |  |
| HciG00081208 | *LPLAT* | lysophosphatidylcholine acyltransferase |
| HciG00033475 | *CK* | choline/ethanolamine kinase |
| HciG00055757 | *SDP* | triacylglycerol lipase SDP1-like |
| HciG00055571 |  |  |
| HciG00082650 | *DPP* | diacylglycerol diphosphate phosphatase |
| HciG00031888 | *GDE* | glycerophosphodiester phosphodiesterase |
