# Supplementary material for: Lipidomic and transcriptomic profiles of glycerophospholipid metabolism during Hemerocallis citrina Baroni flowering
Source: BMC Plant Biol. 2023 Jan 23;23:50. doi: 10.1186/s12870-022-04020-x (PMC9869519; doi:10.1186/s12870-022-04020-x)
Supplement: Supplementary file 4 — Additional file 4: Figure S2. Verification of key genes using qRT-PCR. [file 12870_2022_4020_MOESM4_ESM.docx]

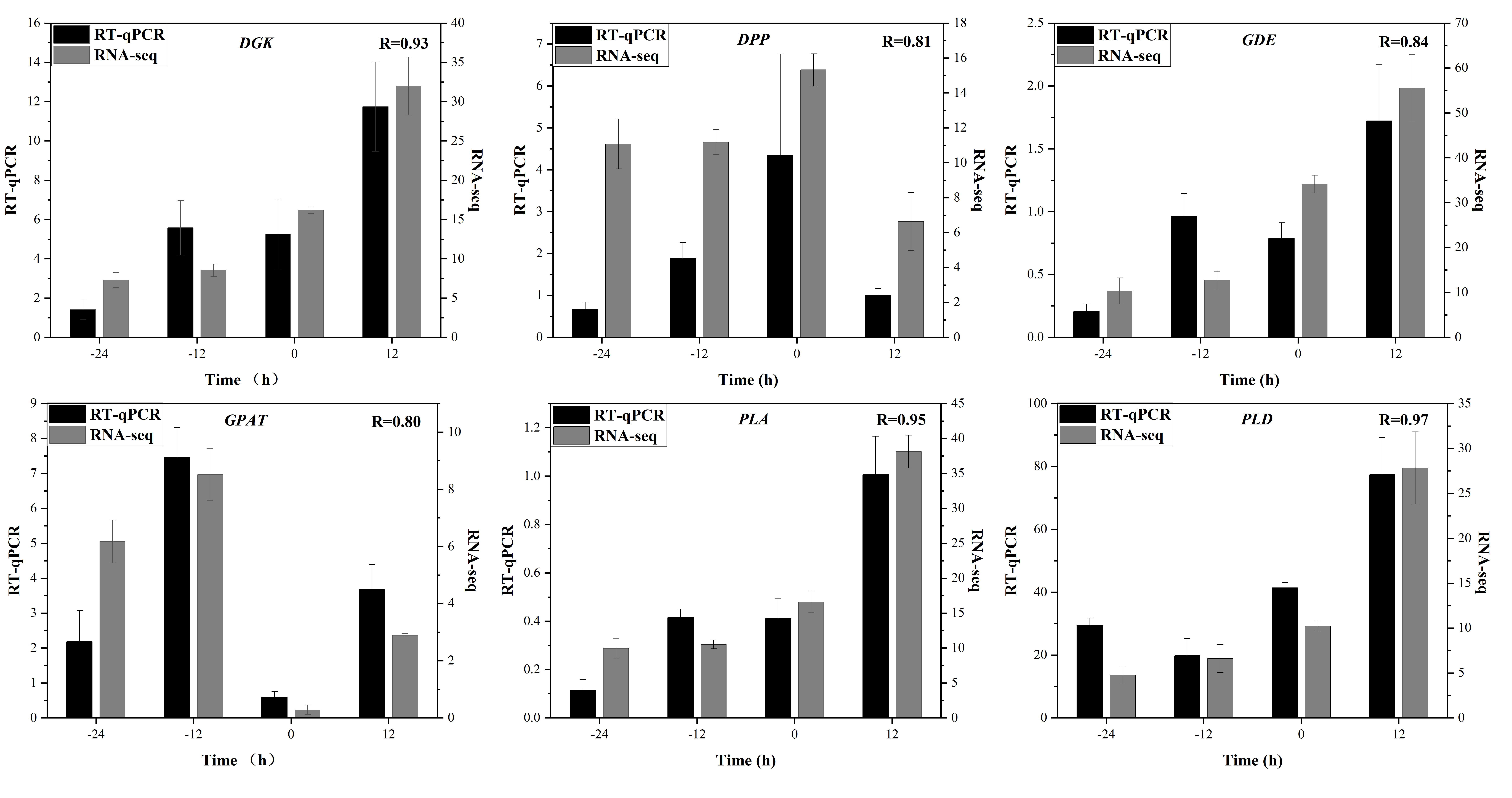


**Figure S2** Verification of key genes using qRT-PCR. Error bars represent standard deviation of three replicates.
