# Supplementary material for: Lipidomic and transcriptomic profiles of glycerophospholipid metabolism during Hemerocallis citrina Baroni flowering
Source: BMC Plant Biol. 2023 Jan 23;23:50. doi: 10.1186/s12870-022-04020-x (PMC9869519; doi:10.1186/s12870-022-04020-x)
Supplement: Supplementary file 5 — Additional file 5: Table S3. Primers of qRT-PCR. [file 12870_2022_4020_MOESM5_ESM.docx]

**Table S3** Primers of qRT-PCR analysis.

| Gene symbol | Forward primers | Reverse primers |
| --- | --- | --- |
| *AP4* | CCGCCTTTACCAATACTCAAGTCC | TCTTGGACCTGCTGCGATGTTTAT |
| *DGK* | GTATCTGAGGAGGGCGATGAAG | CCGCTCTTCGAGTTCACAAA |
| *GPAT* | GCAAGCCTCCTTACATAGAACCA | TGCTAAGTTCCTCGTTATCGCC |
| *GDE* | GAAGAAGCGTTTCAGAAGGTCG | CATCAGGTTGGAAACTCGAGAA |
| *DPP* | CCTGACTTTTTTTGGCGGTG | AGTATGACCACTAGGGAAACTCTTG |
| *PLA* | GGATTTGGTTATGACTTTCGCC | TATCTTTTTGCCCCCGGAG |
| *PLD* | CATAATCATCGGGTCAGCCAAC | TGCGGAAACCGTGAATCTGA |
